# Optimization of the process of preparation of liver pâtés and omental fat of lambs fed with lipid cakes

**DOI:** 10.1371/journal.pone.0304532

**Published:** 2024-07-19

**Authors:** Bruna Almeida da Silva, Vinícius Costa Gomes de Castro, Cleidiane da Silva Araújo, Maria Regina Sarkis Peixoto Joele, André Guimarães Maciel e Silva, Jamile Andréa Rodrigues da Silva, Welligton Conceição da Silva, Ellen Cristina Nabiça Rodrigues, José de Brito Lourenço-Júnior

**Affiliations:** 1 Department of Food Technology, University of the State of Pará, Marabá, PA, Brazil; 2 Graduate Program in Animal Health and Production of the Amazon, Federal Rural University of the Amazon, Belém, PA, Brazil; 3 Graduate Program in Food Science and Technology, Federal University of Pará, Castanhal, PA, Brazil; 4 Federal Institute of Education, Science and Technology of Pará, Castanhal, PA, Brazil; 5 Institute of Veterinary Medicine, Federal University of Pará, Castanhal, PA, Brazil; 6 Federal Institute of Piauí Campus Uruçui, Piauí, PB, Brazil; University of Agriculture in Krakow, POLAND

## Abstract

The aim of this work was to optimize the process of elaborating liver pâtés and omental lamb fat and to evaluate the quality of the products. Livers and fats were obtained from lambs fed with diets composed of corn and soybean meal that were partially replaced by cupuaçu, tucumã and palm kernel cake. To prepare the pâtés, livers were baked for 20 minutes at 100°C, weighed, seasoned, crushed, packaged and pasteurized. The best formulation of the pâté was with 40% liver, 10% fat, 35% water, and pasteurized for 20 minutes at 65°C. The pâté from the livers of lambs fed with palm kernel cake obtained a higher caloric value of 193.05 kcal/100 g and all pâtés met the recommended microbiological quality. There was a significant effect (p< 0.05) of the diets on the aroma and texture of the liver pâtés of lambs fed corn and soybean meal and palm kernel cake, and these were 6.38 and 3.37, respectively. Thus, the pâtés can be considered an alternative to increase the options for consumption of liver from lambs, and also for adding commercial value to lamb viscera.

## Introduction

The ruminant liver has a nutritional value similar to that of some meat cuts, but its consumption is still low, being used in Brazil mainly in the preparation of typical regional dishes such as buchada and sarapatel [1]. Thus, the diversification of the forms of consumption of this viscera can contribute to its valorization by the consumer market [2]. It is worth mentioning that there are also very few products made from ruminant viscera, so it is important to develop research aimed at the production of meat products in order to increase the options for consumers.

Among the derivatives, the pâté is obtained from meats and/or edible kids, from different species of production animals and processed into paste with added condiments and submitted to a suitable thermal process and must contain at least 30% of the raw material which designates it, except for liver, which has a minimum limit of 20% [3].

The feasibility of the use of viscera, processing conditions and nutritional and sensorial quality of liver pâtés has been researched in order to guide the food industry and consumers. The study by Amaral et al. [4] showed that pâté made with sheep liver had a protein content of 15.10 g and 23.90 g of lipids. Martín-Sánchez et al. [5] studying the sensory quality of pork liver pâté observed that the inclusion of alternative ingredients improved the aroma and juiciness, whose mean values were 6.3 and 6.1, respectively, according to the method described in the author’s study.

The relationship between the food product and the consumer is influenced by the sensory characteristics, and several tools are used to optimize, standardize, and develop new parameters to characterize this relationship, as well as to measure the acceptability and the intention to purchase the products [6, 7].

The quality and safety of meat derivatives should be analyzed in order to avoid harmful effects on human health [8]. Thus, pasteurization has been used by industries to ensure food safety through the destruction of pathogenic microorganisms [9–13], but the binomial time and temperature must be applied correctly to avoid drastic changes in products.

Optimizing the process of preparing liver and omental fat pâtés from lambs fed lipid cakes is essential in the quest for efficiency, quality and innovation in the food industry. The need for optimization arises from the growing demand for food products that meet not only regulatory standards. When considering specific ingredients such as lipid cakes in lamb diets, it is crucial to develop a process that maximizes the nutritional benefits of these elements while ensuring the production of consistent pâtés in terms of texture, taste and food safety. Considering consumers’ search for more nutritious, attractive and safe foods, the objective of this work was to optimize the process conditions to obtain pâtés elaborated with liver and omental fat of lambs fed with lipid cakes.

## Material and methods

### Management of lambs

The experiment was conducted at the Federal Institute of Education, Science and Technology of Pará ‐ IFPA (latitude 1° 18’08 "S and longitude 47º56’10" W), after approval of the Ethics Committee on Animal Use ‐ CEUA, Federal University of Pará ‐ UFPA, protocolo nº 8694141217, approved on May 15, 2017.

Twenty-four male Dorper ‐ Santa Inês crossbred lambs, initially with a mean live weight of 30.05 ± 2.45 kg and age between 4 and 5 months were used in this study. The animals were fed for 70 days, with diets consisting of 40% corn silage and 60% concentrate, with partial substitution of corn and soybean meal for cupuaçu, tucumã and palm kernel cake (Table 1).

**Table 1:** Proportion of ingredients and concentrated composition of lamb diets.

		Proportion		
Ingredient	T_1_	T_2_	T_3_	T_4_
Corn silage	40.0	40.0	40.0	40.0
Corn	43.2	6.2	26.0	13.2
Soybean meal	14.8	6.8	14.4	13.9
Cupuaçu cake	-	45.0	-	-
Palm kernel cake	-	-	17.6	-
Tucumã cake	-	-	-	30.9
Mineral-vitamin supplement 1	1.5	1.5	1.5	1.5
Limestone	0.5	0.5	0.5	0.5
Chemical analysis		Diets		
Dry matter (%)	94.56	94.68	95.23	94.73
Organic matter (%)	95.40	94.09	94.89	95.67
Mineral matter (%)	4.34	5.54	4.86	4.20
Crude protein (%)	22.26	19.19	18.83	17.98
Ethereal extract (%)	6.13	8.19	6.65	7.16
FDN	27.24	25.12	33.09	34.99

Treatment ‐ T_1_: Corn and soybean meal; T_2_: Cupuaçu cake; T_3_: Palm kernel cake; T_4_: Tucumã cake. 1 Calcium 140 g, Phosphorus 65 g, Magnesium 10 g, Sulfur 12 g, Sodium 130 g, Cobalt 80 mg, Iron 1000 mg, Iodine 60 mg, Manganese 3.000 mg, Selenium 10 mg, Zinc 5.000 mg, Fluorine (max) 650 mg, Vitamin A 50.000 IU, Vitamin E 312 IU. NDF: Neutral detergent fiber.

The chemical composition of the ingredients of the lambs’ diets was analyzed by determination of dry matter (DM), organic matter (OM), crude protein (CP), ethereal extract (EE), neutral detergent fiber corrected for ash and protein (FDNcp and lignin ‐ LIG, according to INCT-CA [14]. The non-fibrous carbohydrates corrected for ashes and proteins (CNFcp) were calculated according to Detmann and Valadares Filho [15].

After the feeding period, the animals were slaughtered according to the rules of the Industrial and Sanitary Inspection Regulations for Animal Products ‐ RIISPOA [16]. The livers were collected, vacuum packed and identified in: FP ‐ liver of lambs fed corn and soybean meal; FC ‐ with cupuaçu cake; FD ‐ with palm kernel cake and FT ‐ with tucumã cake, and subsequently frozen in a freezer at -18°C.

### Designing and pasteurizing of lamb liver and omental fat

To determine the best proportion of liver, omental fat and water of the pâtés, a design was made up of eleven trials, with eight factorial (defined in 1 and -1) and 3 central (defined in 0). The lowest experimental condition was 26% liver, 10% fat and 35% water; and the largest composed of 40% liver, 20% fat and 53% water and the central condition was 33% liver, 15% fat and 44% water. As an experimental response, the index of acceptability (IA) was evaluated by the tasting-preference test using trained tasters [17], and moisture and pâté lipids were analyzed according to Association of Official Analytical Chemistry ‐ AOAC [18].

After the choice of the pâté formulation, the time and temperature combinations of pasteurization were analyzed from the eleven trials, four factorials (-1 and +1), four axial (-α and + α) and three central (0). The lowest factorial condition consisted of 68°C for 13 minutes and the highest of 82°C for 27 minutes. The axial conditions were 65 and 85° C for 20 minutes and 75° C for 10 and 30 minutes, and the central condition was composed of 75°C for 20 minutes. The values used in the designs were stipulated based on research carried out by Dalmas et al. [19] and Silva et al. [20]. Trial responses were evaluated through the instrumental and sensorial texture of the products. After optimization of the process conditions, the pâtés were elaborated and identified as PP ‐ liver pâté of lambs fed corn and soybean meal; PC–with cupuaçu cake; PD ‐ with palm kernel cake and PT ‐ with tucumã cake, and were characterized according to their physicochemical, microbiological and sensorial analyses.

### Preparation of liver pâtés and omental fat of lambs

The livers were cut and baked for 20 minutes at 100°C, and then cooled to 30° C, weighed and emulsified with omental fat derived from lambs. Ingredients were then added which consisted of 0.3% oregano, 2.3% dehydrated garlic, 2.1% dehydrated onion, 1.8 or 2.0% sodium chloride, 1.5% soy protein, 1.6% monosodium glutamate, 0.8% Hungarian powder, 0.9% Krakoline E, 1.4% urucum dye and 0.3% calabrian pepper, until obtaining a paste and homogeneous mass, which was bottled in glass, pasteurized, cooled and stored at ± 2°C.

### Physicochemical analyses of livers, pâtés and omental fat of lambs

The analyses were performed according to the Association of Official Analytical Chemists ‐ AOAC [18] with humidity analyzed in an oven at 105° C, ash by incineration of organic matter at 550° C, total nitrogen content by the Kjeldahl method, lipids by the Goldfish method, and carbohydrates by the difference between the nutrient values mentioned above, subtracted from 100 [21]. The caloric values of the products were calculated according to RDC 360, dated December 23, 2003 [16].

The pH was determined using a pH meter (Tecnal, TEC-5) previously calibrated with buffer solutions of pH 4 and 7 [22]. The water activity (Aw) was evaluated in an Aqualab apparatus (4TE duo), and color was measured using a colorimeter (Konica Minolta, CR ‐ 410), with reading of the parameters of L * (brightness) ranging from 0 (black) to 100 (white); a * which varies from red (+ a *) to green (- a *), and b * from yellow (+ b *) to blue (- b *). The chromaticity (C *) and the hue angle (h) were calculated according to the equations:

$$C*=\left(a*2+b*2\right)2\;and\;h{}^{\circ}=tan-1\left(b*/a*\right)$$

The instrumental texture of the pâtés was evaluated in a TA-XT Plus (Stable Micro System) texturometer using a cylindrical probe 1.27 cm in diameter and 4.5 cm in length. The hardness of the pâtés packed in glass jars 4 cm in diameter and 5 cm high, with internal temperature of ± 5°C, was analyzed according to Viana et al. [22], with the respective process conditions: pre-test velocity: 1.0 mm/sec, test speed: 5.0 mm/sec, post-test velocity: 10 mm/sec, compression distance: 25 mm, and contact force: 5.0 N.

### Microbiological analyses of liver pâtés and omental lamb fat

Microbiological analysis was carried out after slaughter. The analysis of thermotolerant coliforms was conducted at 45°C using Staphylococcus aureus coagulase, Salmonella and Clostridium sulfite reductive, as recommended by RDC No. 12 of January 2, 2001 [23], according to the methodology described in Normative Instruction IN 62 of 2003 of the Ministry of Agriculture, Livestock and Supply.

### Sensory analysis of liver pâtés and omental lamb fat

The tasters were selected after evaluation of a questionnaire composed of questions about eating habits and health status, applied to 30 students of the Food Technology undergraduate course at the State University of Pará / Campus Castanhal. The tasters were submitted to sensory attributes evaluation tests such as: aroma, flavor and texture, in order to verify the judges’ recognition capacity [17]. Afterwards, the ten testers who scored above 70% of the tests were recruited, with the team composed of individuals of both sexes and ages ranging from18 to 30 years.

Each evaluator received 10 g of pâté served at ± 6°C on traditional toast and used an evaluation card composed of an unstructured scale of 9 cm, with the left of the scale presenting the term "weak or absent" and the right "strong or very", and the judges were instructed to indicate with a vertical line under the scale line the point that best represented the perceived intensity in relation to the attributes analyzed [24]. The overall acceptability of the pâtés was evaluated on a verbal and numerical scale anchored by extremes 1 (very bad) and 5 (excellent). The classification of product acceptability was according to the averages obtained: between 1.0 to 2.9 (unacceptable product); 3.0 to 3.9 (acceptable) and 4.0 to 5.0 (excellent) [25]

### Statistical analysis

The effects of the independent variables on the responses were evaluated by analysis of variance (ANOVA) and the means were compared by the Duncan test at 5% significance, level curve and response surface, and desirability profile with the program Statistica version 7.0. The results of the physicochemical and sensorial composition were evaluated through ANOVA, with means being compared by the Tukey test at a 5% significance level using the SAS program [26].

## Results and discussion

### Physicochemical composition of livers of lambs

The diets did not influence (p > 0.05) ash and protein values, but there was an effect (p < 0.05) on the moisture, lipids, carbohydrates, caloric value and color parameters of lamb livers (Table 2).

**Table 2:** Mean and standard deviation of the physicochemical composition of the liver of lambs.

Analysis	Treatment			
	F_P_	F_C_	F_D_	F_T_
Moisture (%)	69.93 ± 0.26^b^	71.67 ± 0.35^a^	70.01 ± 0.27^b^	71.23 ± 0.34^a^
Ashes (%)	1.68 ± 0.11	1.82 ± 0.10	1.82 ± 0.11	1.63 ± 0.26
Lipids (%)	2.73 ± 0.13^b^	3.43 ± 0.28^b^	3.57 ± 0.54^b^	4.86 ± 0.22^a^
Protein (%)	19.14 ± 0.11	19.51 ± 0.09	19.92 ± 0.15	19.54 ± 0.15
Carbohydrates (%)	6.52 ± 0.14^a^	3.57 ± 0.23^c^	4.68 ± 0.19^b^	2.74 ± 0.27^d^
Caloric value (kcal / 100g)	127.21± 0.18^c^	123.19 ± 0.24^d^	130.53 ± 0.20^b^	132.86 ± 0.18^a^
Brightness	22.42 ± 0.34^b^	23.11 ± 0.22^b^	23.19 ± 0.73^b^	28.28 ± 0.25^a^
Coordinate a *	7.63 ± 0.52^b^	6.32 ± 0.18^c^	8.14 ± 0.48^b^	10.57 ± 0.74^a^
Coordinate b *	6.29 ± 0.13^cb^	5.73 ± 0.47^c^	7.40 ± 0.51^ab^	8.52 ± 0.33^a^

Liver of fed lambs ‐ F_P_: Soybean meal and corn; F_C_: Cupuaçu cake; F_D_: Palm kernel cake; F_T_: Tucumã cake; Means followed by equal letters in the same line do not differ at a significance level of *p* < 0.05.

The moisture of the livers from the diets with corn and soybean meal and palm kernel cake differed (p < 0.05) between the livers of lambs with cupuaçu cake and those with tucumã cake. The moisture found in the livers was propitious to the proliferation of microorganisms, which influences the quality, stability and useful life of this viscera [27]. The results are similar to those reported by Almeida et al. [28] and Lima et al. [2] for lamb liver who showed 74.89% and 74.35% moisture, respectively.

The lipid value of the liver of lambs fed tucumã cake was higher (p < 0.05) and differed from the other treatments. The lipid composition of the tucumã cake may have influenced fat deposition in the liver [29], since according to Enser et al. [30] the lipid amount and profile in the ruminant diet can alter the lipid content of the liver, since this organ metabolizes and stores lipids. These results corroborate those of Lima et al. [2], whose lipid content was 2.62% in liver of lambs fed with sunflower seeds.

The diet with corn and soybean meal increased the carbohydrate value of FP and differed (p < 0.05) from the other analyzed livers. This result is probably due to corn, which contains more soluble carbohydrates than cupuaçu, palm kernel and tucumã. The use of nutrients by ruminants depends on the quality of the ingredients that make up the diet, influencing animal performance and consequently the composition of meat, offal and milk [30, 31].

The results indicate that the inclusion of tucumã cake in the liver diet resulted in a significantly higher caloric value, reaching 132.86 kcal/100 g, which differs in a statistically significant way (p < 0.05) from the other treatments evaluated. This disparity is mainly attributed to the higher lipid content of 4.86% found in FT (presumably one of the treatments). As lipids are the most caloric nutrients, their substantial contribution in FT treatment appears to be the main factor for the significant increase in total liver calories with tucumã cake. These results have important implications for dietary planning, highlighting the need to carefully consider nutritional composition when incorporating specific preparations into the diet, especially for those seeking to control caloric intake or meet specific dietary requirements.

The diet with tucumã significantly increased liver luminosity (p < 0.05) compared to the other treatments. The highest lipid content of 4.86% (Table 2), obtained in the liver of lambs fed with tucumã cake, may have increased the diffusion capacity of light and consequently the luminosity of the liver.

The a * coordinate of FT was superior and tended to be a red color, with a significant effect (p< 0.05) in relation to the other livers. In relation to the b * coordinate, the liver of the lambs fed tucumã cake differed (p < 0.05) from the livers from the diets with corn and soybean meal and with inclusion of cupuaçu cake and tended to the positive axis of the CIE scale which indicates a yellow color, a fact that may be associated with the higher lipid content found in FT (Table 2). In addition, tucumã contains β-carotene and zeaxanthin [32], which are pigments that may have contributed to the a * and b * values of the liver.

### Design of liver pâté and omental fat formulations of lambs

The results obtained in evaluating the acceptability of pâtés reveal a significant variation, with rates ranging between 25.00% and 88.33%. Furthermore, the nutritional composition analysis shows a wide range for moisture (from 59.47% to 72.71%) and lipid (from 9.08% to 21.56%) contents. However, it should be noted that, according to established criteria [33], a food product must have a minimum acceptability index of 70% to be considered satisfactory in terms of its sensory properties. In this context, it is observed that tests 5, 6, 7, 10 and 11 were not well accepted by tasters, as they presented percentages lower than those recommended, indicating significant dissatisfaction in relation to the sensorial characteristics of these pâtés.

The moisture value of test 3 was below the limit recommended by MAPA [34], which establishes humidity of 60% to 70% in pasteurized pâtés, and all the tests presented adequate results for lipid values, according to MAPA [34], whose maximum content should be 32% in pâtés. Meat products that contain saturated fat that should not be consumed in excess, as this is associated with the onset of ailments such as cardiovascular diseases [35]. Thus, among trials 1, 2, 4, 8 and 9, the lowest lipid content of 11.81% associated with the highest acceptability index of 88.33% was found in test 2, showing that this pâté formulation is the most appropriate.

### Design of the pasteurization process of liver pâté and omental fat of lambs

**Fig 1:**
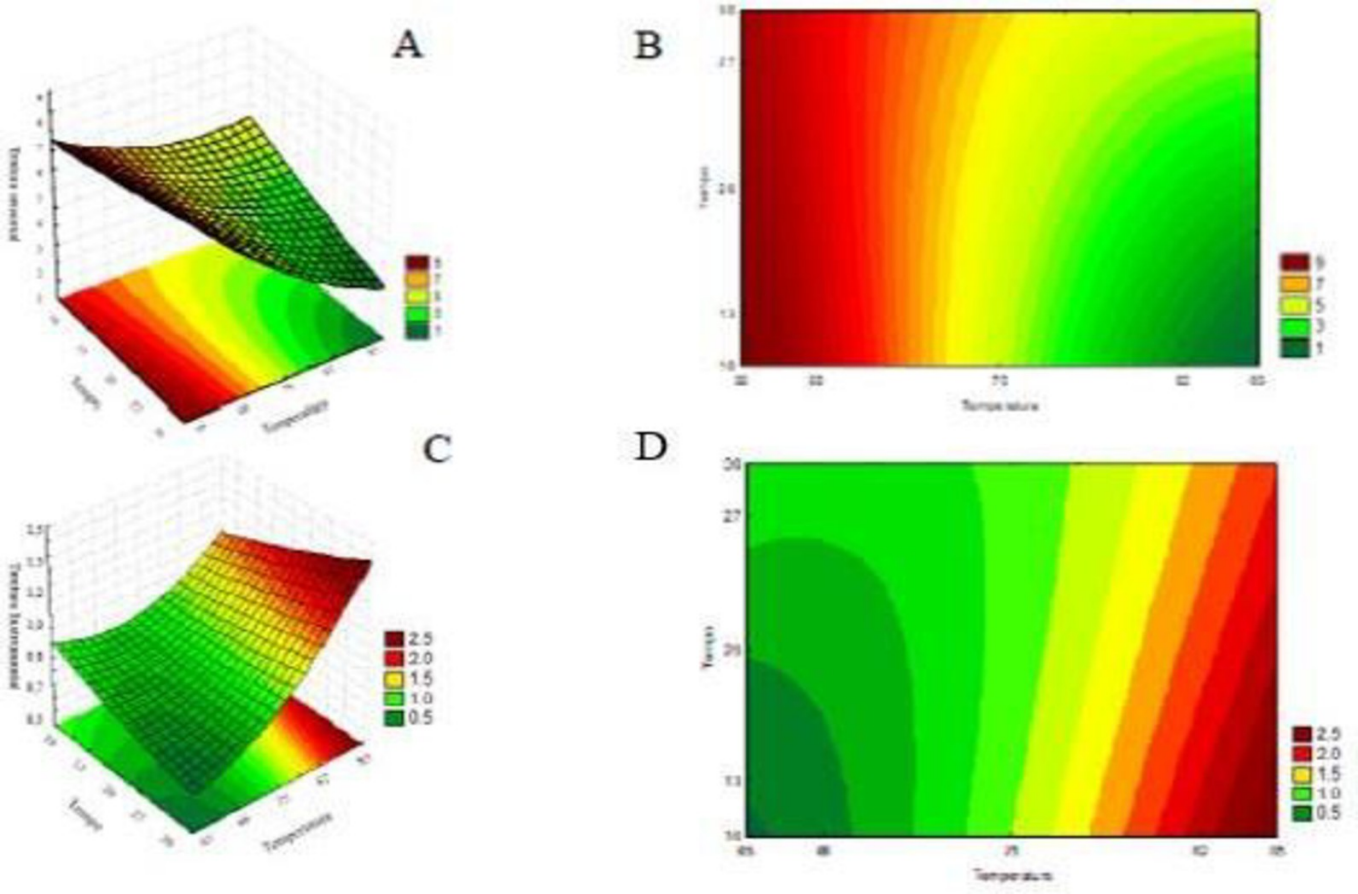
Response surface and level curve. (A–B): Sensory texture as a function of pasteurization; (C–D): Instrumental texture as a function of pasteurization.

The pâté pasteurization design (Table 3), level curve and response surface (Fig 1A and 1B) showed that the technological parameters of pasteurization (Fig 1C and 1D) influenced the texture and consequently the choice of pâté. At higher temperatures, pâtés with stronger instrumental texture were obtained, probably due to the loss of moisture during the process, which may have interfered with the spreadability of the product and the lower acceptability of the sensory texture, as observed in trials 2, 4, 5, 6, 7, 9, 10 and 11. In order for the sensory attributes of a product to be considered acceptable they must present an average of 7 or higher [17], so the pâtés from these tests did not present a desirable texture.

**Table 3:** Pasteurization variables of liver pâtés and omental fat of lambs.

	Independent Variable		Dependent Variable	
Test	Temperature	Time	Instrumental texture	Sensory texture
	(ºC)	(Minutes)	(Newton)	
1	68	13	0.64 ± 0.23^e^	9.1 ± 0.11^a^
2	82	13	1.47 ± 0.01ª	2.8 ± 0.31^c^
3	68	26	0.66 ± 0.26^e^	9.3 ± 0.15^a^
4	82	27	1.12 ± 0.58^b^	5.7 ± 0.35^b^
5	75	20	0.85 ± 0.32^cd^	6.3 ± 0.40^b^
6	75	20	0.93 ± 0.45^c^	5.2 ± 0.38^b^
7	75	20	0.75 ± 0.21^de^	5.5 ± 0.32^b^
8	65	20	0.61 ± 0.24^e^	9.7 ± 0.19^a^
9	85	20	1.46 ± 0.06ª	2.6 ± 0.22^c^
10	75	10	0.81 ± 0.47^cd^	5.2 ± 0.34^b^
11	75	30	0.90 ± 0.50^c^	5.4 ± 0.41^b^

Means followed by equal letters in the same column do not differ at a significance level of *p* < 0.05.

**Fig 2:**
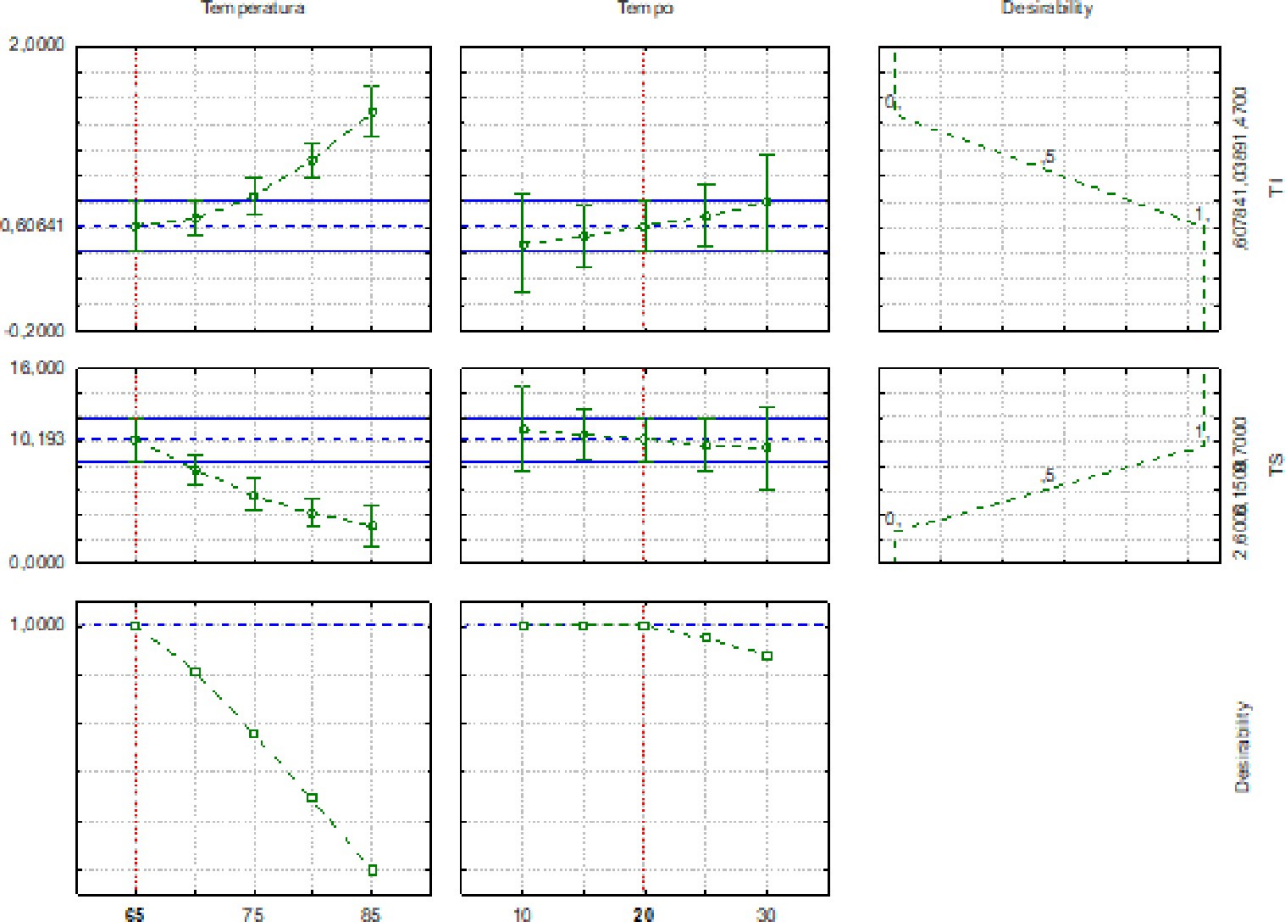
Desirable profile of temperature and pasteurization time of liver pâtés and omental fat of lambs.

The dependent variables in the tests 1, 3 and 8 did not differ (p < 0.05) from each other and presented the lowest values of instrumental texture and the highest means for sensory texture; however the desirability profile (Fig 2) for assay 8 (65°C for 20 minutes) presented the best binomial temperature and pasteurization time of the pâté.

### Physico-chemical composition of liver pâtés and omental fat of lambs

The pâtés did not differ (p > 0.05) in relation to the ash, protein, pH and Aw values. However, moisture, lipids, carbohydrates, caloric value, color and instrumental texture (Table 4) were significant (p ˂ 0.05).

**Table 4:** Mean and standard deviation of the physicochemical composition of liver pâtés and omental fat of lambs.

Analysis	Treatment			
	P_P_	P_C_	P_D_	P_T_
Moisture (%)	62.57 ± 0.76^b^	64.43 ± 0.22^a^	62.82 ± 0.25^b^	64.65 ± 0.10^a^
Ashes (%)	5.53 ± 0.77	4.65 ± 0.43	4.98 ± 0.06	4.99 ± 0.05
Lipids (%)	12.38 ± 0.47^b^	12.96 ± 0.06^b^	12.94 ± 0.01^b^	13.26 ± 0.36^a^
Protein (%)	9.66 ± 0.46	9.81 ± 0.14	9.53 ± 0.39	9.71 ± 0.08
Carbohydrates (%)	9.86 ± 0.21^a^	8.15 ± 0.16^b^	9.73 ± 0.04^a^	7.39 ± 0.13^b^
Caloric value (kcal)	189.52 ± 0.11^b^	188.48 ± 0.24^bc^	193.50 ± 0.12^a^	187.74 ± 0.06^c^
pH	6.45 ± 0.02	6.16 ± 0.01	6.95 ± 0.01	6.56 ± 0.03
A W	0.95 ± 0.01	0.96 ± 0.02	0.97 ± 0.02	0.97 ± 0.01
Brightness	35.49 ± 0.56^b^	37.08 ± 1.72^b^	40.81 ± 1.74^a^	40.47 ± 0.60^a^
Coordinate a *	8.17 ± 0.35^a^	7.09 ± 0.20^b^	7.83 ± 0.31^b^	7.67 ± 0.18^b^
Coordinate b *	20.17 ± 0.60^a^	16.62 ± 0.23^c^	18.12 ± 0.60^b^	18.18 ± 0.43^b^
Croma	21.76 ± 0.67^a^	18.07 ± 0.27^c^	19.74 ± 0.66^b^	19.73 ± 0.44^b^
Tone Angle	67.96 ± 0.44^a^	66.91 ± 0.44^b^	66.62 ± 0.42^b^	67.11 ± 0.46^a^
Texture (N)	0.95 ± 0.23^a^	0.63 ± 0.14^b^	1.01 ± 0.17^a^	0.69 ± 0.19^b^

Liver pâté of lambs fed ‐ P_P_: corn and soybean meal; P_C_: cupuaçu cake; P_D_: Palm kernel cake; P_T_: tucumã cake. Means followed by equal letters in the same line do not differ at a significance level of *p* < 0.05.

The PP and PD moisture were lower (p ˂ 0.05), probably due to the composition of the raw material, since the livers from the lambs fed corn and soybean meal and palm kernel cake had significantly lower values for moisture content. According to MAPA [34], pasteurized pâté must have humidity between 60 and 70%, thus all the formulations are within the limits recommended by the legislation and corroborate the results from Delgado-Pando et al. [36], whose pork liver pâté moisture was 66.18%.

The liver pâté of lambs fed with tucumã cake presented higher lipid content (p ˂ 0.05) than the other pâtés. This result may be associated with the higher lipid content obtained in the liver of lambs fed with tucumã cake (Table 2). Lorenzo et al. [37] mentioned that the amount of added fat interferes with the lipid content of the product, since they found that pâtés with 30% and 40% fat inclusion had a lipid content of 23.2% and 26.6%, respectively, values higher than those obtained in this study, whose inclusion was 10%. According to MAPA [34], pâtés should not exceed 32% of lipids in their composition, so the analyzed products are within this established limit.

The PP and PD showed higher carbohydrate values and differed (p ˂ 0.05) from the other products. It should be noted that the livers from the treatments with corn and soybean meal and palm kernel cake contained the highest values of carbohydrates, a fact that may have influenced the value of this nutrient in pâtés. According to MAPA [34] carbohydrate content can vary from 1 to 10%, so all formulations are within the range considered ideal.

The PD differed (p ˂ 0.05) from PP, PC and PT in energy density, but all results were lower than those found by Terrasa et al. [38], whose caloric values ranged from 288.49 to 416.19 kcal/100 g, in chicken liver pâtés made with different fat proportions.

The highest values of luminosity were obtained in pâtés PD and PT that differed (p ˂ 0.05) from PP and PC. The association of liver moisture, lipids and color of lambs fed with palm kernel cake and tucumã cake may have influenced the refraction of light by the product, and consequently, resulted in greater luminosity. Higher values were found by Amaral et al. [4] in pâté made with liver, blood and sheep meat (42.59), and by Zajac and Swiatek [39], who reported a luminosity of 55.92 in pork liver pâté.

The a* values of the pâtés tended to be red, however, only PP differed (p ˂ 0.05), from PC, PD and PT. Dalmas et al. [19], when analyzing pâté made from liver of lambs, reported that the products with greater value of a * presented lower luminosity, a fact observed in the present work. Furthermore, the highest value of b* and chroma were found in the PP with a significant effect (p ˂ 0.05) compared to the other products, and the higher the value of chroma, the more saturated the color of the product, a fact observed in PP. The PP and PT tone angle differed from PC and PD, as this is a variable that represents the color of the food perceived by the eyes. Thus, the formulations made from corn and soybean meal and tucumã show a greater intensity of the color yellow, since they are closer to the 90° axis.

The PP and PD had the highest values of instrumental texture and differed (p ˂ 0.05) from the other pâtés. This result is due to the lower values of moisture obtained by these products, a fact confirmed by Delgado-Pando et al. [36] whose highest Fc of 2.36 N was derived from the 50.08% lower moisture formulation in pork liver pâtés.

### Microbiology of liver pâtés and omental lamb fat

The microbiological results of the pâtés were thermotolerant coliforms ˂ 3.10 NMP/g, absent of Salmonella spp and Coagulase positive *Staphylococcus* and *Clostridium* sulfite reductant ˂1.10 CFU/g. Thus, the pâtés presented a microbiological quality that can be considered safe and fit for consumption, since they met the values established by Resolution 12 of the National Health Surveillance Agency [23].

### Sensory attributes of liver pâtés and omental lamb fat

No significant effect (p > 0.05) was observed for the appearance attributes (color and presence of lumps), flavor (fat and seasoning), taste (liver, fat, salty) and texture (adhesiveness) of pâtés (Table 5).

**Table 5:** Mean and standard deviation of the sensory attributes of liver pâtés and omental fat of lambs.

Attribute	Treatment			
	P_P_	P_C_	P_D_	P_T_
**Appearance**				
Color	3.53 ± 0.08	4.56 ± 0.99	5.29 ± 0.94	4.04 ± 0.14
Presence of lumps	2.84 ± 0.94	2.37 ± 0.65	2.72 ± 1.87	2.23 ± 0.96
**Aroma**				
Liver	6.38 ± 0.35	5.39 ± 0.77	5.11 ± 0.98	5.23 ± 0.73
Fat	4.52 ± 0.18	4.33 ± 0.11	4.81 ± 1.80	3.99 ± 0.39
Spice	3.27 ± 0.62	2.96 ± 0.68	2.62 ± 1.75	3.06 ± 0.20
**Flavor**				
Liver	5.44 ± 0.72	4.82 ± 0.84	4.94 ± 0.52	5.30 ± 0.23
Fat	6.69 ± 0.81	6.40 ± 0.29	6.44 ± 0.31	5.17 ± 0.12
salty	3.61 ± 0.38	3.08 ± 0.18	3.13 ± 0.00	3.63 ± 0.47
**Texture**				
Tougzhness	2.79 ± 0.15^b^	1.71 ± 0.19^c^	3.37 ± 0.23^a^	1.13 ± 0.88^c^
Adhesiveness	1.80 ± 0.03	1.23 ± 0.92	1.80 ± 0.31	1.17 ± 0.56
Overall acceptability	3.30 ± 0.01	3.50 ± 0.02	3.20 ± 0.03	3.50 ± 0.02

Liver pâté of lambs fed ‐ P_P_: Soybean meal and corn; P_C_: Cupuaçu cake; P_D_: Palm kernel cake; P_T_: Tucumã cake. Means followed by equal letters in the same line do not differ at a significance level of *p* < 0.05

According to the tasters, the hardness of the liver pâté of lambs fed with palm kernel cake was superior and differed (p ˂ 0.05) from PP, PC and PT. This result can be related to the value of the instrumental texture and moisture of the product, since PD presented higher values (1.01 N) and humidity (62.82%; Table 6). The results corroborate with Amaral et al. [4] whose sensory texture of pâté elaborated with sheep liver was 7.1. It should be noted that despite the statistical difference in aroma and texture of the products, the overall acceptability ranged from 3.20 to 3.50, and according to Meilgaard et al. [25] products with averages ranging from 3.00 to 3.90 are considered acceptable.

**Table 6:** Variables of the formulations of liver pâtés and omental fat of lambs.

	Dependent Variable (%)		
Test	IA	Moisture	Lipids
1	75.00 ± 0.02^cd^	66.32 ± 0.45^b^	12.62 ± 0.31^d^
2	88.33 ± 0.43^a^	66.18 ± 0.31^b^	11.81 ± 0.35^d^
3	77.78 ± 0.52^bc^	59.47 ± 0.79^c^	21.56 ± 0.54^a^
4	85.00 ± 0.23^ab^	60.29 ± 0.52^c^	19.30 ± 0.48^b^
5	25.00 ± 0.11^f^	72.71 ± 0.62^a^	10.06 ± 0.81^e^
6	51.19 ± 0.32^e^	72.24 ± 0.61^a^	9.08 ± 0.73^e^
7	43.33 ± 0.67^e^	65.76 ± 0.40^b^	18.10 ± 0.65^b^
8	73.81 ± 0.71^d^	67.09 ± 0.39^b^	15.86 ± 0.34^c^
9	70.37 ± 0.22^cd^	65.49 ± 0.31^b^	14.97 ± 0.54^c^
10	66.67 ± 0.56^cd^	67.03 ± 0.47^b^	14.56 ± 0.21^c^
11	68.52 ± 0.32^cd^	66.71 ± 0.36^b^	14.82 ± 0.36^c^

IA: Acceptability index; Means followed by equal letters in the same column do not differ at a significance level of *p* < 0.05.

## Conclusion

The optimized process conditions for pâté production were 40% liver, 10% fat, 35% water, with pasteurization at 65°C for 20 minutes. Under these conditions, the physicochemical and microbiological properties of the liver pâtés of lambs fed cupuaçu, palm kernel cake and tucumã cake were desirable, since they met the standards of current legislation and presented sensorial quality. Thus, the elaborated pâté can be considered to be an alternative to increase the options of sale and consumption of lamb liver, besides adding commercial value to the viscera from the slaughter of ruminants.
